# Reduction of Brain Mitochondrial β-Oxidation Impairs Complex I and V in Chronic Alcohol Intake: The Underlying Mechanism for Neurodegeneration

**DOI:** 10.1371/journal.pone.0070833

**Published:** 2013-08-13

**Authors:** James Haorah, Travis J. Rump, Huangui Xiong

**Affiliations:** Neurovascular Oxidative Injury Laboratory, Department of Pharmacology and Experimental Neuroscience, University of Nebraska Medical Center, Omaha, Nebraska, United States of America; University of Windsor, Canada

## Abstract

Neuropathy and neurocognitive deficits are common among chronic alcohol users, which are believed to be associated with mitochondrial dysfunction in the brain. The specific type of brain mitochondrial respiratory chain complexes (mRCC) that are adversely affected by alcohol abuse has not been studied. Thus, we examined the alterations of mRCC in freshly isolated mitochondria from mice brain that were pair-fed the ethanol (4% v/v) and control liquid diets for 7–8 weeks. We observed that alcohol intake severely reduced the levels of complex I and V. A reduction in complex I was associated with a decrease in carnitine palmitoyltransferase 1 (cPT1) and cPT2 levels. The mitochondrial outer (cPT1) and inner (cPT2) membrane transporter enzymes are specialized in acylation of fatty acid from outer to inner membrane of mitochondria for ATP production. Thus, our results showed that alterations of cPT1 and cPT2 paralleled a decrease β-oxidation of palmitate and ATP production, suggesting that impairment of substrate entry step (complex I function) can cause a negative impact on ATP production (complex V function). Disruption of cPT1/cPT2 was accompanied by an increase in cytochrome C leakage, while reduction of complex I and V paralleled a decrease in depolarization of mitochondrial membrane potential (ΔΨ, monitored by JC-1 fluorescence) and ATP production in alcohol intake. We noted that acetyl-L-carnitine (ALC, a cofactor of cPT1 and cPT2) prevented the adverse effects of alcohol while coenzyme Q10 (CoQ10) was not very effective against alcohol insults. These results suggest that understanding the molecular, biochemical, and signaling mechanisms of the CNS mitochondrial β-oxidation such as ALC can mitigate alcohol related neurological disorders.

## Introduction

Alcohol is abused/used by millions of Americans, and is responsible for many loss of lives, physical disfigurations and broken family throughout the world [1]. In deed, misuse of this legalized substance of abuse is an urgent concern to global health problem. Unfortunately, there is no known effective drug to prevent alcoholism or its damaging effects. A daily nutrient supplement in food intake may hold beneficial strategy to alleviate the deleterious health effect among heavy chronic alcohol users. Achievement of this goal requires understanding the underlying mechanisms of alcohol-elicited cellular/tissue damage. Alcohol-induced tissue-organ or organelle injury is mediated via the metabolism of ethanol by alcohol dehydrogenase and cytochrome P450 2E1 that produce acetaldehyde as the primary metabolite [2], [3], [4]. The uniqueness of CYP2E1 mediated ethanol metabolism is that it produces acetaldehyde and reactive oxygen species (ROS) as primary metabolites. Thus, tissue injury or toxicity in the brain will occur in those cell types that express the ethanol metabolizing or the radical generating enzymes. We have shown the induction of CYP2E1 by ethanol in human brain endothelial cells, astrocyte, neurons and macrophage [5], [6], [7], [8]. Recently, Jin et al. (2013) demonstrated the regulation of CYP2E1 expression and oxidative stress-mediated signaling pathway in astrocytic and monocytic cell lines [9]. However, we were the first to discover that activation of free radical generating enzymes NADPH oxidase, inducible nitric oxide synthase and mitochondrial damage by acetaldehyde exacerbates the production of ROS and reactive nitrogen species in the brain [5], [6]. Therefore, ethanol is a producer (via CYP2E1) and an inducer (via acetaldehyde) of oxidative stress.

Alcohol-induced oxidative stress end-organ injury [2], [7], [10] is believed to be the results of mitochondrial damage [11], [12], [13], [14], [15] and decrease of ATP production [16]. Mitochondrial damage and subsequent oxidative production are known to initiate the development of neurological diseases [17], [18]. Key factors involved in alcohol-elicited mitochondrial dysfunction may arise from altering the outer and inner mitochondrial membranes that regulate the transport of energy substrates via β-oxidation. Defective mitochondrial β-oxidation may imbalance oxidative phosphorylation, leading to leakage of oxidative products from the matrix and depletion of ATP in the brain. The CNS neurovascular cells utilize glucose as the main energy source. But alcohol causes hypoglycemia, thereby limiting the availability of glucose for ATP synthesis in the brain. We demonstrated the mechanisms that alcohol inhibits the transport/metabolism of glucose in the brain by affecting the glucose transporter 1 [19], [20]. Therefore, improving the glucose metabolic pathway and stabilizing the carnitine palmitoyltransferase-1 and -2 (cPT1/cPT2) mediated β-oxidation of high-energy generating pathway are necessary for neuronal protection in alcoholics.

We demonstrated that physiological relevant concentration of ethanol (0.1 %) elevated the levels of reactive oxygen species and nitric oxide via the induction of NADPH oxidase and inducible nitric oxide synthase in primary human neurons that were accompanied by marked oxidative damage and neuronal loss [5]. Interestingly, acetyl-L-carnitine (ALC, a mitochondrial energy substrate transporter) significantly prevented these effects, suggesting a direct involvement of defective mitochondrial β-oxidation and oxidative phosphorylation in alcohol-induced neuronal degeneration. ALC contains the carnitine essential for fatty acids acylation and acetyl moiety that maintains the acetyl-CoA level essential for acetylcholine production and neurotransmission in the brain [21]. Thus, ALC is a delivery form of carnitine and acetyl group in clinical therapeutic setting, in which carnitine palmitoyltransferase-1 and -2 (cPT1 and cPT2) transport fatty acids into the mitochondria for bio-fuel production. ALC is known to restore mitochondrial decay function in aging [22] as well as a neuroprotective agent in stroke and Alzheimer's disease [23], [24]. ALC has been shown to increase choline acetyltransferase activity (cholinergic neuronal function) in rat hippocampus [25].

We hypothesized that stabilization of mitochondrial outer (cPT1) and inner (cPT2) membrane function by ALC can stabilize the efficient supply of substrates for oxidative phosphorylation, thereby, maintains the matrix oxidant-antioxidant balance for improved ATP production in alcohol abuse. Thus, we propose that administration of ALC can improve mitochondrial β-oxidation, restore antioxidant levels, replenish ATP generation, and improve neurotransmission in neurological disorders impacted by substance of abuse such as alcohol. The significance of these findings is that timely therapeutic intervention of ALC can improve mitochondrial energy regulation, antioxidant level, repair enzyme activity, and prevention of neurological diseases.

## Materials and Methods

### Animal

To evaluate the EtOH-induced mitochondrial damage, five weeks old male mice (C57BL/6J; from Jackson Laboratory, Bar Harbor, ME, USA) were acclimated to 1–4% EtOH liquid-diet or control liquid-diet for 5 days prior to pair-feeding regimens of control or 28% ethanol caloric intake (4% wt/vol, or 870 mM) liquid-diets for 7–8 weeks. Pair feeding was based on the amount of food consumed by ethanol group animal. Experimental conditions consisted of 1) control liquid diet, 2) ethanol (EtOH) liquid diet, 3) ALC in EtOH liquid diet, and 4) CoQ10 in EtOH liquid diet using 12 mice per condition. Macronutrient composition of control-liquid diets (Lieber–DeCarli diets from Dyets Inc.) as percent of total calories is 47% carbohydrate, 35% fat, and 18% protein; whereas, the EtOH-liquid diet is 35% fat, 18% protein, 19% carbohydrate and 28% ethanol caloric intake. Doses of ALC and CoQ10 were 1.0 mg each/mL liquid diet. Daily food intake and weekly body weight were recorded, while blood samples were collected at the time of sacrifice for determination of alcohol level. Mice were euthanized by intraperitoneal injection of 0.2 ml ketamine (100 mg/ml or 800 mg/kg) at 7–8 weeks. Brain tissues from 6 mice/condition were used for protein extraction and tissue sections. Remaining brain tissues from 6 mice/condition were used for isolation of mitochondria.

### Brain tissue homogenates and mitochondria isolation

Using Mitosciences Isolation Kit (Mitosciences, Eugene, Oregon), mitochondria were isolated from mice brain tissues (without cerebellum) weighing about 0.28–0.35 g. Briefly, brain tissues were minced, followed by 35 Dounce strokes to rupture the tissues in a pre-chilled Dounce homogenizer containing 2.0 ml of isolation buffer. Homogenates were centrifuged at 1,000×g for 10 min at 4°C, discard pellets (tissue fibers) and collect supernatants as fraction **A** (whole brain homogenates). Further centrifugation of fraction **A** at 12,000×g for 15 min at 4°C yielded fractions **B** (homogenates with minimal mitochondria) and **C** (mitochondrial fraction). Fraction **C** was washed two times with 1.0 ml of isolation buffer and reconstituted in 500 μl of isolation buffer (contains protease inhibitor cocktail) as freshly isolated mitochondrial fraction. Enrichment of mitochondrial complex I – V was assessed in fractions **A–C** by Western blot using antibody to mitochondrial complex I – V (Mitosciences, Eugene, Oregon). As expected fraction **C** was highly enriched with mitochondrial complex I – V but not fractions **A** and **B**. Thus, isolated mitochondrial fraction **C** was used for the assays of JC-1 membrane potential and ATP production. Aliquots were stored at −80°C for alterations of mitochondrial complexes by Western blot.

### JC-1 membrane potential

The assay is based on the changes in electrochemical gradient across the mitochondrial membrane measured by membrane potential (MP). Loss of MP is detected by a fluorescent cationic dye JC-1 (5,5′,6,6′-tetra chloro-1,1′,3,3′-tetraethylbenzamidazolo-carbocyanin iodide (Cell Technology, Mountain, CA). Briefly, freshly isolated mitochondria were labeled with JC-1 for 15 min at 37°C in a 5% CO2 incubator in tubes. After two washes with assay buffer, JC-1 labeled and unlabeled mitochondria suspensions were transferred to a 96-well black wall plates. Fluorescence intensity was read at excitation 550 nm, emission 600 nm for red fluorescence and at excitation 485 nm, emission 535 nm for green fluorescence using an M5E fluorescence plate reader. The ratio of red to green determined the rate of cells dead. A decrease in the ratio indicated dying of cells.

### Cytochrome C release

Cytochrome C release was assayed by human cytochrome C titerzyme enzyme immunometric assay (EIA) kit (Assay Designs, Ann Arbor, MI). This kit uses a monoclonal antibody to cytochrome C immobilized on a microtiter plate to bind cytochrome C in the standards or samples (40 μg lysates protein). Excess samples or standards are washed out after 1 hr incubation at 37°C, followed by addition of biotinylated monoclonal antibody to cytochrome C. The antibody bound to cytochrome C was captured on the plate. Excess antibody was washed out and streptavidin conjugated to alkaline phosphatase was bound to biotinylated monoclonal cytochrome C antibody following 30 min incubation. After washing out the excess conjugate, the substrate enzyme was incubated for 45 until the reaction was stopped and the color formation was read at 405 nm with correction between 570 and 590 nm. Results were calculated after subtracting average blank OD value from that of mean standards or samples OD values. The concentrations of cytochrome C in the samples were determined by interpolation from standard curve.

### ATP production

Production of ATP in freshly isolated mitochondria or in cell culture was determined by Molecular Probes ATP determination kit (Eugene, OR) and by following the method of Drew and Leeuwenburgh [26]. ATP standard curves were run in the range of 0.125 to 16 μM (0.125, 0.25, 0.5, 1.0, 2.0, 4.0, 8.0, and 16.0 μM). The optimum reaction mixture was maintained at 28°C, and the luciferase assay for ATP production was performed on fluorescence plate reader with luminometer function (M5, Molecular Devices, Sunnyvale, CA) using 96 well plates in triplicates for each condition. ATP level was normalized to mitochondrial protein concentration.

### Ex-vivo electrophysiology

Long-term potentiation synaptic neurotransmission was studied in *ex-vivo* frontal cortical brain tissue slice of chronic alcohol intake and control mice. Briefly, 4 groups of mice (4 mice per group) consisting of control, EtOH, EtOH+ALC, ALC alone were fed the control or 28% ethanol caloric intake (4% wt/vol, or 870 mM) liquid diets for 6 weeks containing the same macronutrient composition of Lieber–DeCarli diets as described above. ALC dosage (2.0 mg/gm body weight) was administered daily for 7–8 wks, which was mixed in liquid diets. Brains were removed from the cranial cavity and were placed into ice-cold pre-oxygenated artificial cerebrospinal fluid (ACSF). Transverse brain slices (400 μm in thickness) prepared from frontal cortex were kept in a humidified/oxygenated holding chamber at 24°C for 1 hr prior to transferring the brain slices into the recording chamber. The detail procedures of low-frequency stimulus (LFS) and long-term potentiation (LTP) synaptic transmission recording in *ex-vivo* brain tissue slices have been described previously [6].

### Immunofluorescence and microscopy

Frozen brain tissue sections (8 μm thickness) in glass slides were washed with PBS, fixed in acetone-methanol (1∶1 v/v) fixative, blocked the cellular antigen with 3% bovine serum albumin at room temperature for 1 hr in the presence of 0.4% Triton X-100 and incubated with respective primary antibodies such as mouse anti-cPT1/ant-cPT2 (1∶250 dilution) and rabbit anti-neurofilament (1∶250 dilution) for overnight at 4°C. After washing with PBS, tissue sections were incubated for 1 hr with secondary antibody: anti-mouse-IgG Alexa fluor 488 for cPT1/cPT2 and anti-rabbit-IgG Alexa fluor 594 for neurofilaments. Cover slips were then mounted onto glass slides with immunomount containing DAPI (Invitrogen), and fluorescence microphotographs were captured by fluorescent microscopy (Eclipse TE2000-U, Nikon microscope, Melville, NY) using NIS elements (Nikon, Melville, NY) software.

### Statistical analysis

Values are expressed as the mean ± SD. Within an individual experiment, each data point was determined from three to five replicates. Statistical analysis of the data was performed by using GraphPad Prism V5 (Sorrento Valley, CA). Comparisons between samples were performed by two-way ANOVA with Dunnett's post-hoc test. Differences were considered significant at P values ≤0.05.

## Results

**Figure 1:**
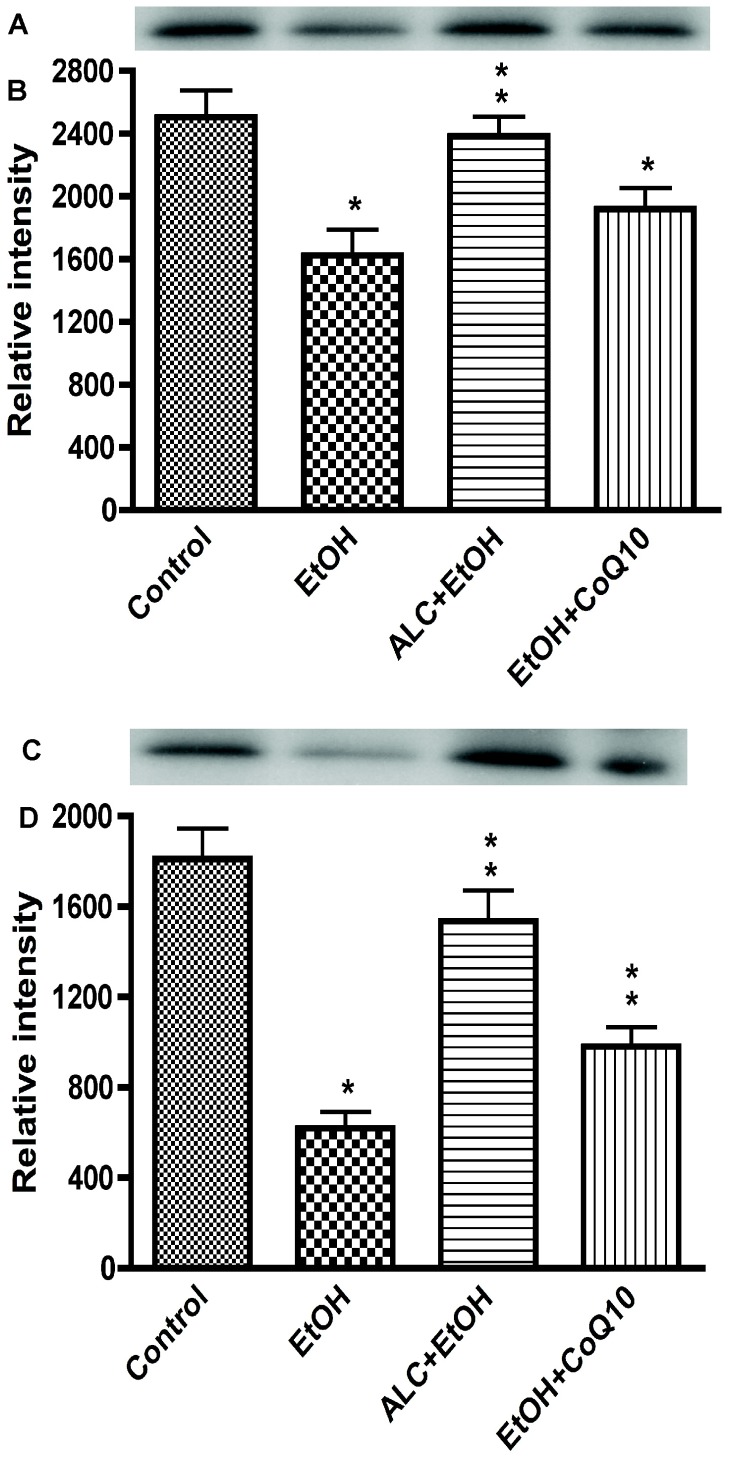
Freshly isolated mitochondria from frontal cortex of mice pair-fed the control or ethanol liquid-diets for 7–8 weeks were examined for alterations of mitochondrial complexes. (A) Representative immunoreactive bands and (B) relative intensity of immunoreactive bands of complex I. (C) Representative immunoreactive bands and (D) relative intensity of complex V immunoreactive bands. Results are expressed as mean values ± SD; n = 6. Statistical significance (p<0.01) is indicated by asterisk compared with controls or double asterisks compared with EtOH condition.

We examined the alterations of mitochondrial complexes in fraction **C** (purified mitochondrial fraction). Mitochondrial complex V (ATP synthase) appeared to be the most affected followed by complex I (NADH-ubiquinone oxidoreductase) in chronic alcohol intake **(** **Fig. 1** **A–D)**. Although, complex II -IV were also altered to some extent, changes in these complexes were not as significant as those of complex V and I. Thus, in the present studies, the internal loading controls were not used for Western blot analyses for two reasons. One, it was not ideal to use any of this complex as an internal standard for normalization of mitochondrial complex I and V because there was some changes occurred in complex II – IV. Second, a purified mitochondrial samples were used for Western blot experiments, as such it was not ideal to use actin as an internal standard controls. We also observed that acetyl-L-carnitine (ALC) prevented the alcohol-induced reduction in mitochondrial complex I and V, but not by CoQ10. ALC was used here to improve mitochondrial β-oxidation, restore antioxidant levels, replenish ATP generation, and improve neurotransmission. These results suggested that alcohol could have impaired the mitochondrial electron transport chain complex I at the substrate entry compartment, thereby affecting the conversion of ATP in complex V in the CNS. Identifying the specific subunit of complex V that is targeted by ethanol is an ongoing research interest.

**Figure 2:**
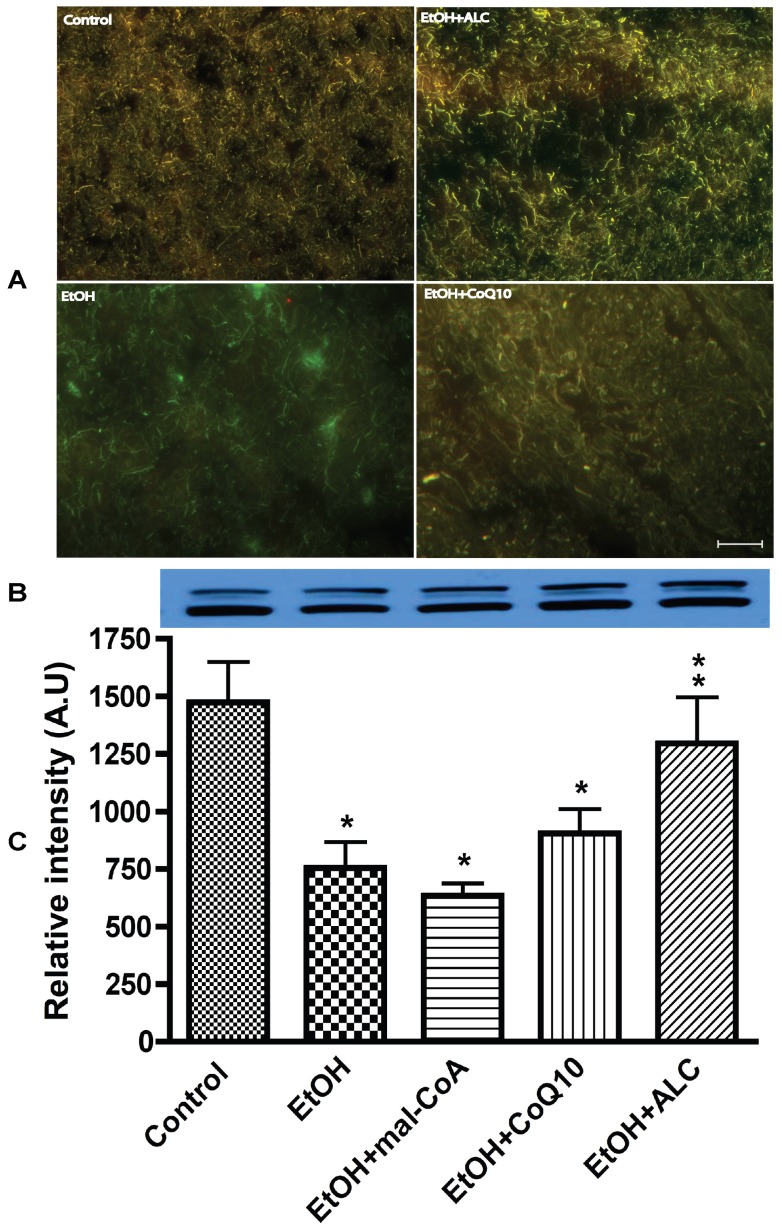
Acetyl-L-carnitine preserves the integrity of mitochondrial outer membrane protein cPT1 from chronic alcohol intake in mice. (A) cPT1 protein colocalization with neurons in brain tissue sections. Neuronal marker protein neurofilament (green) and cPT1 (red). Scale bar indicated 10 μm and original magnification is ×20. (B) Representative immunoreactive bands and (C) quantitative immunoreactive intensity of cPT1 in purified mitochondria. Results are expressed as mean ± SD; n = 6. Statistical significance (p<0.01) is indicated by asterisk compared with controls or double asterisks compared with EtOH condition.

We examined the idea that impairment of mitochondrial membranes substrate transporter enzymes such as carnitine palmitoyltransferase 1 and 2 could be responsible for the break down of mitochondrial respiratory complex I. Thus, we analyzed the mitochondrial membrane transport enzyme cPT1 (outer) and cPT2 (inner) in mice brain tissue sections and in mitochondria fraction **C**. We found that cPT1 protein expression was lower in alcohol condition than the controls and cPT1 appeared to co-localize with neuronal cell bodies in brain frontal tissues sections as indicated by neurofilament staining and confocal microscopy analysis (**Fig. 2** **A**). This observation was validated by Western blot in mitochondria fraction **C** in which co-administration of ALC clearly protected the integrity of cPT1 dimmers from chronic alcohol insults (**Fig. 2** **B–C**). Administration of malonylcoenzyme A (mal-CoA), a specific inhibitor of beta-oxidation was expected to exacerbate the effect of alcohol on cPT1/2 reduction. It did appear to diminish cPT1 protein level slightly, but statistically it was not significant. CoQ10 (an electron carrier from complex I and II to complex III) was used here for stabilization of mitochondrial oxidative phosphorylation during alcohol insults, but it did not show much protective effect on cPT1 protein. We suggest that the non-protective effect of CoQ10 could be attributed to the insolubility of this compound in body fluid.

**Figure 3:**
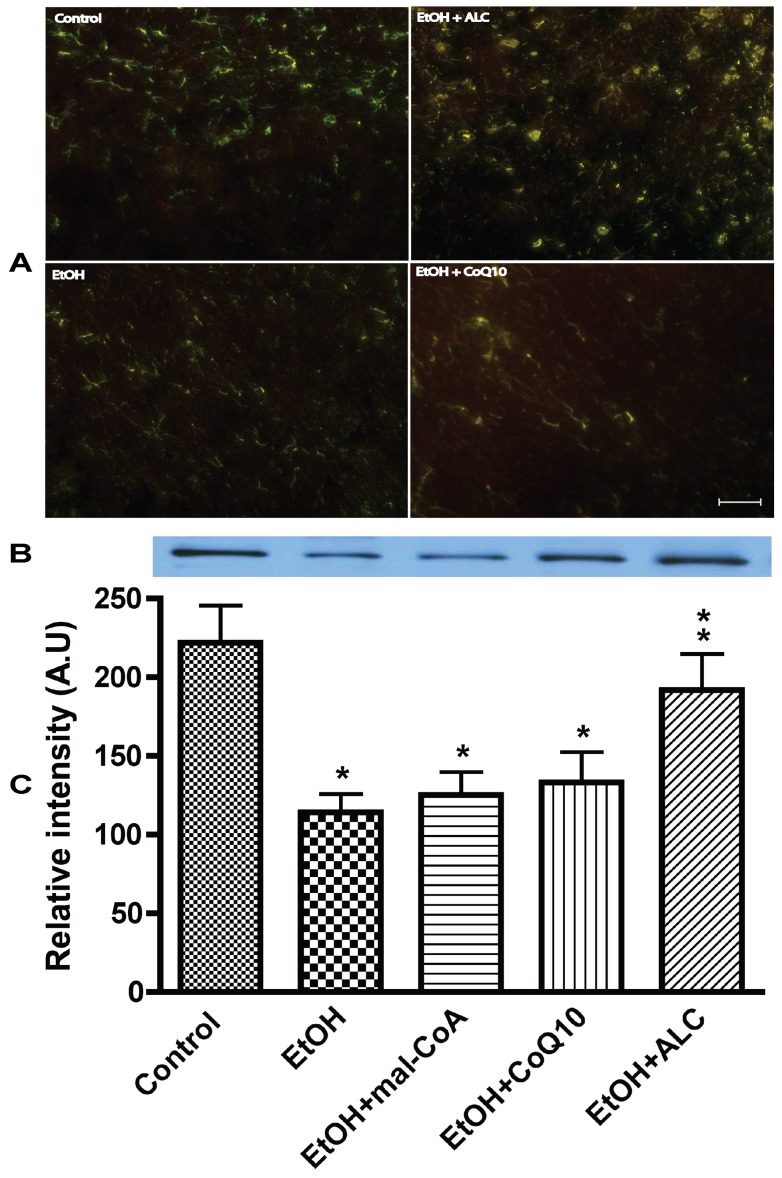
ALC protects the inner membrane protein cPT2 integrity from chronic alcohol ingestion. (A) Expression of mitochondria inner membrane specific cPT2 protein in frontal cortex tissue sections, in which cPT2 protein (red) is colocalized with neurofilament (green). Scale bar indicated 10 μm and original magnification ×20. (B) Representative immunoreactive bands and (C) quantitative immunoreactive intensity of cPT2 in purified mitochondria. Results are expressed as mean ± SD; n = 6. Statistical significance (p<0.01) is indicated by asterisk compared with controls or compared with EtOH (double stars).

We then analyzed the changes in the inner membrane transporter carnitine palmitoyltransferase-2 (cPT2) in brain tissue sections and isolated mitochondria. It was evident that cPT2 protein was expressed in neurons as demonstrated by the co-localization cPT2 and neurofilament protein (**Fig. 3A**). Unlike cPT1, we detected a single band cPT2 monomer protein in all conditions, but much lower than cPT1 protein level (**Fig. 3** **B–C**). Administration of ALC convincingly protected the integrity of cPT2 protein from alcohol insults, whereas, CoQ10 or mal-CoA afforded little or no protection of cPT2 protein level. Taken together, these data indicate that cPT1 and cPT2 are actively expressed in the CNS, as such brain cells may likely utilize β-oxidation of fatty acids as an alternative energy source during alcohol stress. Disruption of mitochondrial cPT1) and cPT2 is likely to cause cytochrome C release since cytochrome C is localized between the outer and inner membranes of the mitochondria. Thus, we assayed the cytochrome C release in fraction **B** as a potential marker for activation of an intrinsic apoptotic pathway.

**Figure 4:**
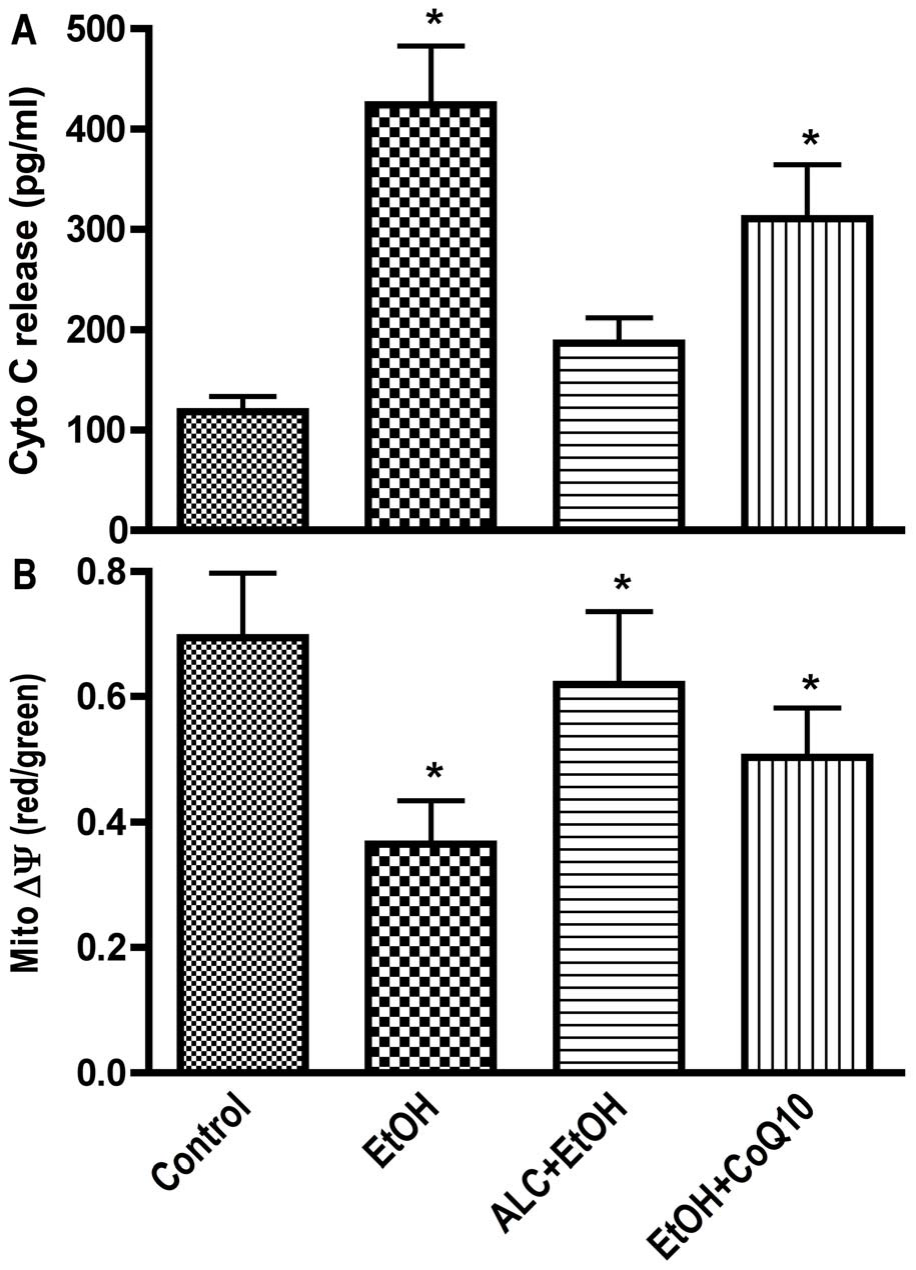
Freshly isolated mitochondria were assessed for changes in membrane potential by JC-1 red and green fluorescence assay and Cytochrome C release by titerzyme enzyme immuno-metric assay kit. (A) Changes in mitochondria membrane potential by JC-1 in which the ratio of red to green determines the rate of membrane potential decay and cells dead. (B) Cytochrome c release. Results are expressed as mean values ± SD; (n = 6). Statistical significance (p<0.01) is indicated by asterisk compared with controls or compared with EtOH (double stars).

We found that chronic alcohol ingestion significantly increased cytochrome C release in mice brain compared with pair fed controls (**Fig. 4A**). ALC significantly prevented the alcohol-induced cytochrome C release, while mal-CoA or CoQ10 did not significantly prevent cytochrome C release from alcohol insults. These data suggest that binding of cytochrome C to apoptotic protease-activating factor 1 (Apaf-1) could be possible mechanisms for activation of caspase-9 and caspase-3 leading to the subsequent cell apoptosis in chronic alcohol intake. Changes in mitochondrial membrane potential (ΔΨ) are important predictor of disruption of mitochondrial function (complex I and V presented in figure 1) and an indicator of cell apoptosis (result of cytochrome C). Thus, we assessed the depolarization of mitochondrial ΔΨ by JC-1 fluorescence in freshly isolated mitochondria fraction **C**. Our results indicated that reduction in ΔΨ activity by alcohol was effectively protected by ALC, while mal-CoA or CoQ10 administration did not restore the membrane potential (**Fig. 4B**). JC-1 once enters into mitochondria reversibly changed color from green to red in viable cells thus increases the mitochondrial ΔΨ by forming J-aggregates complex. JC-1 remains in monomeric form showing intense green fluorescence in dying cells.

**Figure 5:**
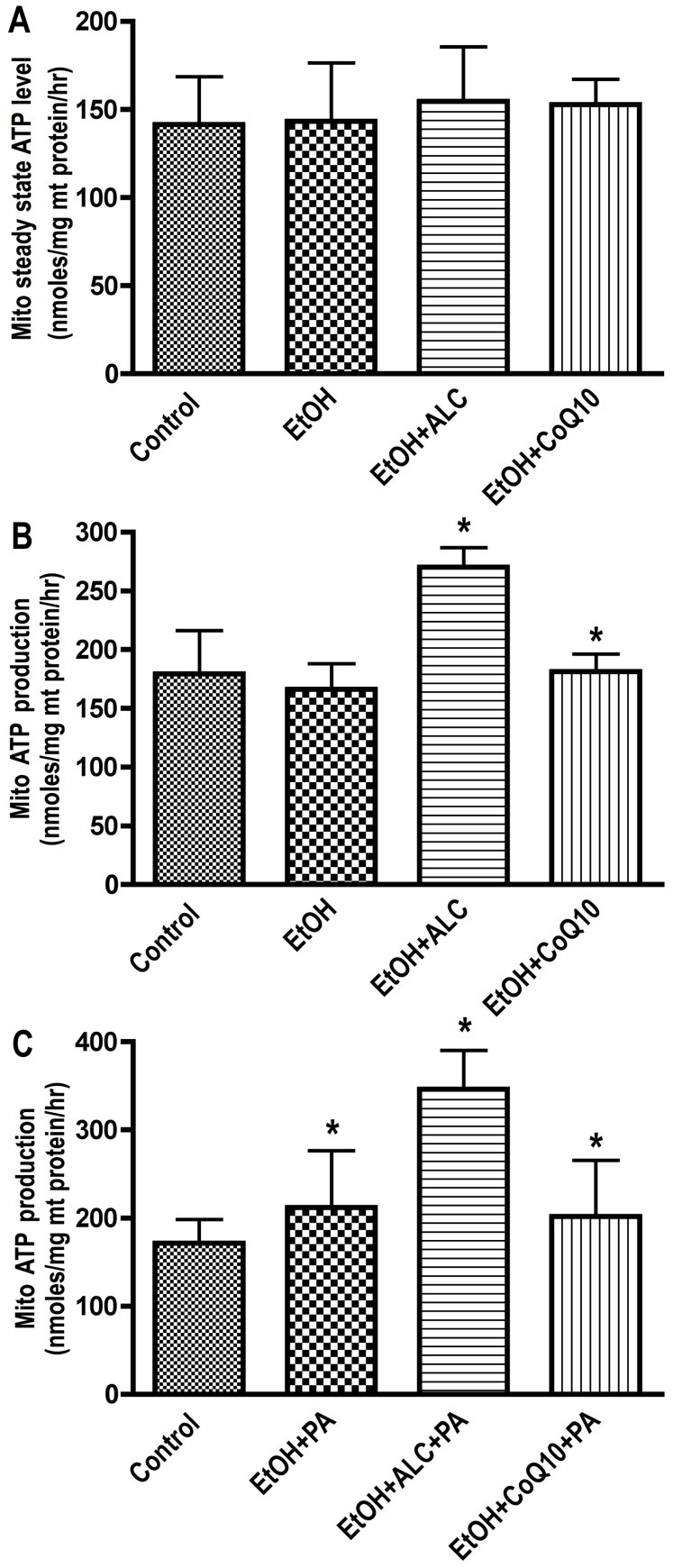
Mitochondria isolated from 7–8 weeks pair-fed the control/EtOH liquid-diets with/without ALC or CoQ10 mice brain tissues were assayed for ATP level by luminescence SpectraMax M5 microplate reader. (A) Steady-state level of ATP in isolated mitochondria from respective groups. (B) ATP production in freshly isolated mitochondria in respective groups following supplementation of ALC or CoQ10 (0.1 mM each) to purified mitochondrial fractions. (C) Utilization of palmitic acid (PA) in isolated mitochondria in respective experimental conditions after supplementation of ALC or CoQ10 during PA oxidation. The levels of ATP were calculated from ATP standard curve run in parallel. Results are expressed as mean nmoles/mg protein ± SD; (n = 6). Statistical significance (p<0.01) is indicated by asterisk compared with controls.

Complex V (ATP synthase) was the worst affected compartment in the respiratory chain by chronic alcohol intake. We proposed this effect to be an outcome of cPT1/cPT2 and complex I alterations. This is because blockade of substrate entry and disruption of electron transfer is expected to imbalance the oxidative phosphorylation and energy production. To evaluate the breakdown of substrate entry process and ATP converting complex, we analyzed the steady-state level of ATP and efficiency of palmitate (PA) oxidation in freshly isolated mitochondrial in the presence or absence of ALC. The steady-state level of ATP in fraction **C** was not changed without the supplementation of substrate (PA) or stimulation of mitochondrial activity by ALC (**Fig 5** **A–C**). However, exogenous addition of ALC to freshly isolated mitochondria significantly elevated the rate of ATP production in EtOH+ALC or in control, but not in ethanol alone (**Fig 5** **B**). These results suggest that ALC was able to preserve the mitochondrial of β-oxidation of fatty acid machinery, particularly the cPT1 and cPT2 integrity from the damaging effect of alcohol insults. To convince this notion, we determined the oxidation of PA in isolated mitochondria with/without ALC. Our results showed a significant increase in the rate of PA utilization (indicated by ATP production) in mitochondria from control or EtOH+ALC compared with EtOH alone or EtOH+CoQ10 condition (**Fig. 5** **C**). These findings suggest that ALC in deed protected the integrity of mitochondrial β-oxidation machinery and that of ATP converting complex. These data further supported the argument that brain cells are capable of utilizing fatty acids as alternative source of energy during alcohol-induced stress condition.

**Figure 6:**
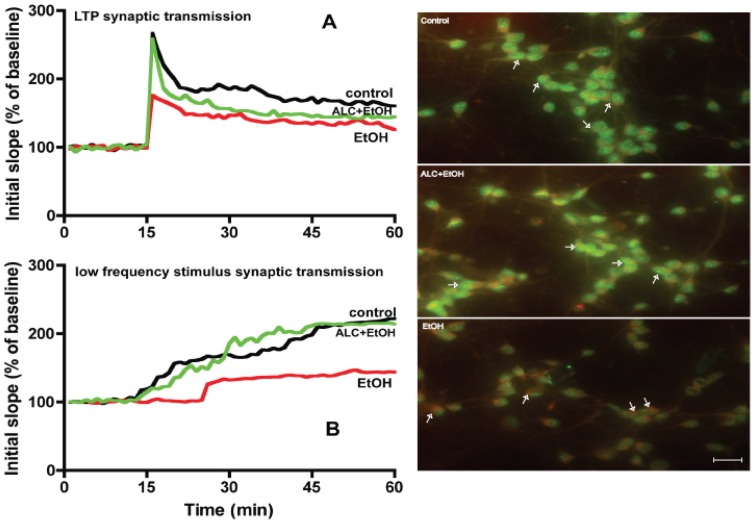
ALC prevents the alcohol-induced reduction of LTP and LFS synaptic transmission in the CA1 region of mice hippocampal slices. *(A)* shows time course and LTP synaptic responses induced by a constant current stimulation (twin pulses, 150–300 μA, 40 μs, 0.05 Hz) of Schafer collateral fibers in three respective conditions. The graph plots the initial slope of the first evoked excitatory postsynaptic potentials (EPSPs) recorded from the CA1 dendrite field (stratum radium) in response to constant current stimuli. *(B)* Illustrates the LFS synaptic transmission in the CA1 region of mice hippocampal slices. Control (**Black**), EtOH (Red), EtOH+ALC (Green), and results were expressed as mean values (± SD; n = 3). **Right panels:** Alcohol impairs mitochondria outer and inner membrane proteins. The marker proteins were probed with antibody to monoamine oxidase A+B (outer membrane marker, Green) and antibody to H6/C12 (inner membrane marker, Red) from Abcam (Cambridge, MA). Conjugated primary antibody was detected by fluorescence Alexa Fluor 488 anti-mouse or Fluor 555 goat-anti chicken secondary antibody. Scale bar indicated 10 μm and original magnification ×40.

We then tested the idea that this mitochondrial dysfunction and energy depletion observed in this chronic alcohol intake could be a possible mechanism for impairment of synaptic neurotransmission. This rationale was logical because neurotransmission is a highly energy driven process. Thus, we rationalized that ALC can be a suitable protective agent to stabilize mitochondrial energy production as well as enhance the neurotransmission in alcohol abuse. ALC can impact such dual functions since ALC is capable of donating carnitine group for improvement of β-oxidation and energy production, and ALC can donate acetyl group for enrichment of acetylcholine (neurotransmitter) in the brain. As expected, brain slices from alcohol intake mice exhibited a significant reduction in long-term potentiation (LTP) and low frequency stimulus (LFS) synaptic transmission, while ALC protected the LTP and LFS synaptic neurotransmission in CA1 region compared with controls (**Fig. 6** **A–B**). Prevention in alcohol-elicited loss of LTP and LFS synaptic transmission by ALC in the frontal cortex suggest that ALC was able to protect the neurons in this region. We speculate that ALC protected neurons by stabilizing the mitochondrial function within this cell type, which validate our previous findings that ALC protects primary human neurons from alcohol insult [5]. This functional change was in part related to qualitative alterations of mitochondria membranes in primary neuronal culture. Alterations of mitochondria outer membrane marker (monoamine oxidase A+B) and inner membrane marker (H6/C12) in neuronal culture were examined by immunohistochemistry and microscopy using respective chicken polyclonal antibody to monoamine oxidase A+B and mouse monoclonal antibody to H6/C12 (Abcam, Cambridge, MA). Treatment of neuronal culture with 20 mM EtOH altered the localization of both the outer and inner membrane proteins compared with control, while ALC effectively protected the disruption of mitochondrial membranes (**Fig. 6,** right panels). Since Co-Q10 was not effective in protecting mitochondria from alcohol and mal-CoA did not exacerbate the effect of alcohol on mitochondria β-oxidation, we only focused the protective effect of ALC on synaptic neurotransmission and mitochondrial membrane function.

## Discussion

**Figure 7:**
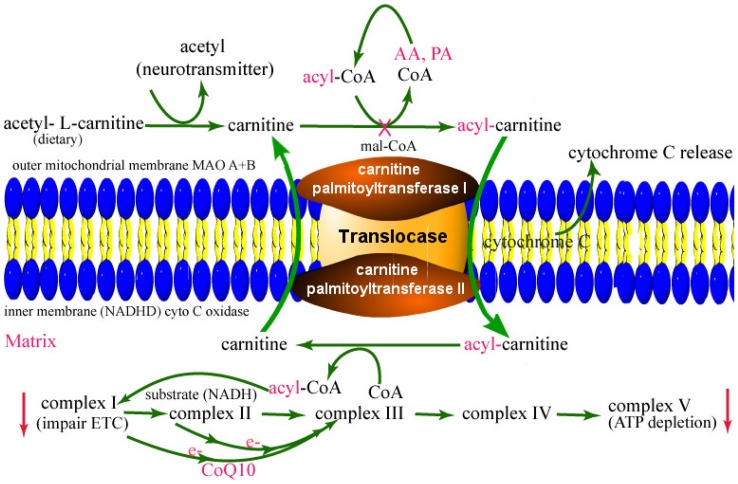
Summarizes the significance of the present findings in a schematic pathways.

The present findings revealed that impairment of mitochondrial membrane transport enzymes (cPT1 and cPT2) involved in oxidation of high energy yielding fatty acid was likely to cause the disruption of complex I and V in alcohol abuse. Protection of this mitochondria β-oxidation of fatty acid could stabilize the proper maintenance of membrane potential, oxidative phosphorylation, sufficient energy production, and ATP-driven neurotransmission in alcohol abuse. The significance and interconnection of the present findings are summarized in figure 7 schematic pathways. Brain cells use glucose as the primary source of energy and ketone bodies as secondary source of energy in physiological condition. This metabolic pathway is altered during alcohol intake because alcohol causes hyperglycemia at low doses and hypoglycemia after chronic ingestion. Thus, the availability of glucose in the brain is limited during chronic alcohol ingestion. The mechanisms of alcohol-induced hyperglycemia and hypoglycemia in the brain remain elusive. Recently, we demonstrated that inhibition of glucose transporter 1 (GLUT1) at the interface between the blood and the brain affects the transport and metabolism of glucose in the brain [19], [20]. Inhibition of glucose entry into the brain is likely to cause hypoglycemia in the CNS and hyperglycemia in the circulation. Both of these clinical conditions can impair glycolytic pathway, in which neuroimmune cell adapts to alternative metabolic pathway such as the β-oxidation of fatty acid. Most recent finding by Taub et al. (2013) makes our present discovery more excitingly significant, in which they reported that inhibition of glycolysis significantly enhanced β-oxidation of fatty acid (1.7–4.5-fold) in non-activated CD4 memory T cells in human subjects [27]. Thus, studies aim at improving the glucose transport or improving the β-oxidation of fatty acid in the CNS is necessary for bio-fuel production during alcohol abuse. Our findings indicated that ALC is effective in improving the mitochondrial β-oxidation, oxidative phosphorylation, and ATP-driven neurotransmission in mouse model of chronic alcohol ingestion. We showed that ALC is neuroprotective by promoting antioxidant activity, enriching acetylcholine transferase, and restoring the β-oxidation function in alcohol exposure [5], [6]. As such, ALC is used as an effective therapeutic agent for neurological complication like Alzheimer's disease [21].

The present studies identified that chronic alcohol ingestion affected three interconnected components of the mitochondria: the membrane substrate transport function (cPT1 and cPT2), the substrate entry site (complex I), and the ATP production site (complex V) in the electron transport chain. Dysfunction of this individual component is well known in human metabolic disease, thus cPT1 knockout mice model is used a model for obesity and inborn errors of mitochondrial β-oxidation [28], [29], whereas, defective complex I and complex V is a signature of neurological diseases such as Parkinson's and Alzheimer's [21], [30]. Interestingly, reduction in complex I and V was implicated for fast tract aging process, as demonstrated in animal model of aging [31]. The underlying mechanisms of cPT1/2 and complex I/V reduction observed in the present study is implicated to oxidative/nitrosative damage since defective mitochondria is a primary site for ROS production [30], [32]. Argument for cPT1/2 dysfunction is supported by recent findings that metabolism of arachidonic acid or palmitic acid in the presence of ethanol decreases membrane potential/ATP levels and increase ROS production in isolated liver mitochondria [33]. This finding suggest that due to defect in cPT1 and cPT2 mediated β-oxidation, these long chain fatty acids are channelized to eicosanoids production to promote mitochondrial injury in the development of alcoholic fatty liver disease. Similarly, ethanol was shown to decrease oxygen consumption in isolated mouse brain mitochondria [34], which indicates an impairment of complex I/V in the respiratory chain. Thus, oxidative or nitrosative damage at 8 kDa subunit of complex I and alpha subunit of the complex V have been implicated as useful biomarkers for neurodegenerative disorders. Similarly, tyrosine nitration of cPT1 was reported in suckling rat cardiac mitochondria [35].

In these studies, ethanol concentration of 4% resulted a blood alcohol level of 0.07–0.13%, which is lower than 0.31% detected in moderate to severely intoxicated alcohol abusers [36], [37]. This moderate concentration of ethanol was enough to damage the mitochondrial membrane function and certain specific respiratory chain complexes. Disruption of mitochondrial membrane function was evident by the alterations of cPT1/cPT2, mitochondrial membrane potential and cytochrome C release. Unlike this finding, Frier et al. recently reported that consumption of a high-fat diet did not increase cPT1 mRNA level [38]. This was expected because high concentration of fatty acid/lipid in fact inhibits the mitochondrial β-oxidation of fatty acid machinery. Alteration of respiratory complex was indicated by the reduction in mitochondrial complex I and V. The isolated mitochondria in these studies were obtained from all brain cell types as such it will be of interest in future to identify the cell type in which these mitochondria are mostly affected by alcohol intake. In agreement with the disruption of substrate entry step and ATP converting complex, chronic alcohol intake decreased the steady-state level of ATP in the whole brain homogenates (fractions A and B) without much changes in fraction C in the absence of exogenous substrate supplementation. It is possible that the steady-state level of ATP is mostly localized outside the mitochondria (fractions A and B), which may not reflect the dynamic function of mitochondria in the absence of substrate supply. Thus a decrease in steady-state ATP level would suggest the breakdown of ATP producing machinery such as complex V. There was no significant change in the steady-state level of ATP between the pair-fed controls and ethanol diet intake in fraction C, which did not reflect the mitochondrial damage by alcohol. The explanation to this anomaly is that most of the ATP is utilized for fueling the oxidative phosphorylation and less ATP is secreted as steady-state in the absence of exogenous substrate. This rationale was justified by the oxidation of palmitate in fraction C, where mitochondria derived from controls/ethanol+ALC produced ATP much higher than ethanol intake during PA oxidation. These results suggested that ALC protected cPT1 and cPT2 for efficient mitochondrial β-oxidation of fatty acid as well as complex V function for effective production of ATP from the chronic alcohol ingestion.

CoQ10 transfers electrons from complex I and II to complex III, which is crucial step to create proton pump gradient across the membrane for generating ATP by complex V. Thus, CoQ10 acts as an energy carrier and as a naturally occurring antioxidant in the mitochondria by a unique mechanism known as the oxidation-reduction cycle. CoQ10 gets reduced as it accepts electrons and it becomes oxidized as it gives up electrons. This unique oxidation-reduction state of CoQ10 prevents the generation of hydroxyl and lipid peroxyl radicals thereby prevent lipid peroxidation and derailment of oxidative phosphorylation in the mitochondria. It has been reported recently that high concentration of ethanol caused the depletion of CoQ10 with concomitant increase in TNF-alpha secretion in HepG2 cell line [39]. However, unlike ALC, we found that exogenous supplementation of CoQ10 afforded less protective effect on mitochondrial function in the CNS. Our in vitro data indicated that CoQ10 was hardly permeable across the cell and mitochondrial membranes, which may not attract as a suitable therapeutic agent for CNS disorders.
